# Vision loss in patients with giant cell arteritis treated with tocilizumab

**DOI:** 10.1186/s13075-021-02480-4

**Published:** 2021-03-22

**Authors:** Jennifer Amsler, Iveta Kysela, Christoph Tappeiner, Luca Seitz, Lisa Christ, Godehard Scholz, Odile Stalder, Florian Kollert, Stephan Reichenbach, Peter M. Villiger

**Affiliations:** 1 Department of Rheumatology, Immunology and Allergology, Inselspital, Bern University Hospital, University of Bern, Bern, Switzerland; 2 Department of Ophthalmology, Inselspital, Bern University Hospital, University of Bern, Bern, Switzerland; 3 Clinical Trial Unit (CTU), Inselspital, University of Bern, Bern, Switzerland

**Keywords:** Giant cell arteritis, Tocilizumab, Glucocorticoids, Anterior ischemic optic neuropathy (AION), Vision loss

## Abstract

**Objectives:**

Giant cell arteritis (GCA) may lead to vision loss. To what extent tocilizumab (TCZ) is able to prevent vision loss is unknown. The aim was to analyze the occurrence of vision loss in a large GCA cohort treated with TCZ.

**Methods:**

In this observational monocentric study, GCA patients treated with TCZ between the years 2010 and 2018 were studied. Demographic, clinical, and laboratory data were analyzed.

**Results:**

A total of 186 patients were included (62% female); 109 (59%) fulfilled the American College of Rheumatology (ACR) criteria, in 123 (66%) patients, large vessel vasculitis was diagnosed by magnetic resonance-angiography (MRA). Cumulative duration of TCZ treatment was 224 years, median treatment duration was 11.1 (IQR 5.6–17.9) months. Glucocorticoids (GC) were tapered over a median of 5.8 (IQR 3.0–8.5) months. At baseline, visual symptoms were present in 70 (38%) and vision loss in 21 (11%) patients. Patients with vision loss at baseline were older (*p* = 0.032), had a lower C-reactive protein (*p* = 0.002), and showed a negative association with MRA of the aorta (*p* = 0.006). Two patients (1.1%) developed vision loss, both at the initiation of TCZ treatment.

**Conclusion:**

Our data show a very low incidence of vision loss in TCZ-treated patient. The two cases of AION occurred at the initiation of therapy, they support the hypothesis that advanced, and established structural changes of arteries are key factors for this accident. Whether a shorter duration of concomitant GC treatment is risky regarding vision loss needs to be studied.

## Key messages

- Vision loss is a rare event during therapy with tocilizumab
- The data suggest a comparable incidence for tocilizumab and for glucocorticoid therapy
- The early events support the hypothesis that advanced structural changes with lumen narrowing contribute to the risk for vision loss

## Introduction

Giant cell arteritis (GCA) is the most common vasculitis in Western populations at older age [1, 2]. Vision loss caused by ischemic events of the posterior ciliary arteries of the ophthalmic artery (anterior ischemic optic neuropathy, AION) or of the central retinal artery (central retinal artery occlusion, CRAO) is one of the most feared complication [3]. To revert it, glucocorticoids (GC) are prescribed immediately [4]. However, most often it remains irreversible.

The rate of vision loss in patients with GCA seems to have decreased over the last decades, probably due to earlier diagnosis of GCA and prompt start of GC treatment [5]. Nevertheless, a recent retrospective study showed a prevalence of 2% of vision loss in 840 biopsy-proven GCA compared to the reference population of Skane (Sweden) with a prevalence of 0.6% [6]. Further studies have documented vision loss mainly due to AION during treatment with GC at a variable rate between 0.7 to 10% [7–10].

IL-6 plays a central role in the pathogenesis of GCA [11]. Accordingly, tocilizumab (TCZ), a monoclonal antibody targeting the IL-6-receptor, was studied in the treatment of GCA. In addition to a remission-maintaining efficacy, the first two randomized controlled trials (RCTs) documented a steroid-sparing effect of approximately 50% compared with a conventional treatment with GC over 1 year [12, 13]. While no ocular incidences were recorded in the first trial [12], one of 149 patients in the GiACTA trial suffered from AION while under TCZ treatment in the first 12 months [13]. So far, no larger study has addressed the question, whether TCZ prevents vision loss comparable to GC monotherapy.

Therefore, we analyzed the frequency of vision loss in a large cohort of patients treated with TCZ and evaluated potential risk factors for vision loss.

## Patients and methods

Data of 186 patients with GCA treated with TCZ between 1st January 2010 and 31st December 2018 at the Division of Rheumatology and Clinical Immunology of the University Hospital (Inselspital) Bern, Switzerland, were extracted from patient charts and entered in a REDCap database, which was prepared for this study and hosted at the Clinical Trial Unit (CTU) of the University of Bern, Switzerland. REDCap (Research Electronic Data Capture) is a secure, web-based software platform designed to support data capture for research studies. The patients fulfilled the criteria for GCA as defined in the two previously published RCTs [12, 13], i.e., patients either fulfilled the American College of Rheumatology (ACR) criteria of GCA and/or they suffered from symptoms of polymyalgia rheumatica (PMR) plus large vessel vasculitis (LVV) as diagnosed by magnetic resonance angiography (MRA).

Baseline was defined as the time of diagnosis of GCA. Changes in vision loss were assessed by determination of best corrected visual acuity [14]: Amelioration was defined as gain of two or more Snellen lines on the visual acuity chart and deterioration as loss of two or more lines on the visual acuity chart. Relapse was defined as the re-occurrence of disease activity attributable to active inflammation that was followed by an increase in GC treatment [4].

### Statistical analysis

All analyses were done using Stata 15 (Stata Corporation, College Station, Texas). We compared baseline characteristics of patients with and without vision loss prior to baseline, using the chi-squared test and the Wilcoxon rank-sum test as appropriate. We displayed the median durations of follow-up, tocilizumab, glucocorticoids, and concomitant treatments. The patient-years of tocilizumab treatment were also computed. Counts of visual impairment and vision loss during the follow-up were displayed. A number of relapses were also recorded and displayed regarding treatment time. We compared baseline characteristics of patients with and without relapses during follow-up, using the chi-squared test and the Wilcoxon rank-sum test as appropriate. Association between permanent vision loss at baseline and the following baseline characteristics: age, first CRP, jaw claudication, and abnormal MRA aorta status were shown in a table and estimated with a multivariate logistic regression model with all the variables presented in the table. Due to the low number of outcome, a limited number of covariates (maximum of 2 to 4) could be included in the model. PMR was not included, because its difference was not significant in the crude comparison between the two groups. We excluded fever from this model because there were no patients with fever and vision loss before baseline. Crude and adjusted odds ratio for all the other characteristics were computed, their 95%-confidence intervals and *p* value were displayed.

### Ethical approval

The cantonal ethical board of Bern, Switzerland, has approved this retrospective study. All patients gave their written general informed consent for the evaluation of their data.

## Results

### Patient characteristics

A total of 186 patients diagnosed with GCA were treated with GC and TCZ according to published RCTs [12, 13], i.e., treatment was started with prednisone (PDN) at a dose of 1 mg/kg body weight per day or three pulses of intravenous corticosteroid treatment depending of the ocular involvement followed by 1 mg/kg body weight of PDN. TCZ was added intravenously in doses of 8 mg/kg bodyweight at 4-weekly intervals or at a dosage of 162 mg subcutaneously at weekly or bi-weekly intervals. 18 patients received a 3-day pulse of 500 or 1000 mg methylprednisolon. Median duration of PDN treatment was 7.7 (IQR 5.2; 12.0) months with a concomitant treatment duration with tocilizumab during tapering of PDN to 0 mg/day of 5.8 (IQR 3.0; 8.5) months; median duration of TCZ therapy was 11.1 (IQR 5.6; 17.9) months with tapering of TCZ during the last months. 72/186 (39%) patients started TCZ within 1 month after diagnosis. For the 114 patients who started TCZ after 1 month mean duration was 11.3 (21.7) months (SD), median duration was 3.5 [1.5;10.8] months [IQ-range].

**Fig. 1:**
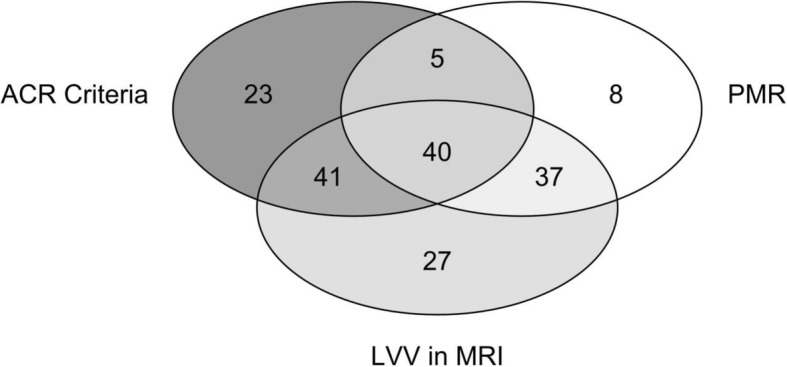
Venn diagram showing number of patients fulfilling the different diagnostic criteria

Patient characteristics at baseline are summarized in Table 1 and displayed in Fig. 1 in a Venn diagram. A total of 109 (59%) patients fulfilled the ACR criteria for GCA, 145 (78%) the criteria used in recent RCTs, i.e., vasculitis based on histology and/or imaging methods [12, 13]. Four of the patients categorized as PMR had a positive histology in temporal artery biopsy, one an AION and one a positive PET-CT, two were diagnosed as PMR-associated GCA based on elevated ESR/CRP, age, and after exclusion of differential diagnoses.

**Table 1:** Baseline characteristics

	All
	*n* (%) or median (IQ range)
Total *N*	*N* = 186
Female	116 (62%)
Age at diagnosis	71.0 (63.0; 77.0)
Weight [kg]	70.0 (59.0; 83.4)
BMI [kg/m^2^]	24.9 (22.0; 28.4)
CRP (mg/L)	50.0 (20.0; 99.0)
ESR (mm/h)	70.0 (40.0; 86.3)
ACR Criteria 1990	109 (59%)
Cranial symptoms (including visual symptoms)	124 (67%)
Visual symptoms	70 (38%)
Vision loss	21 (11%)
Headache	93 (50%)
Jaw claudication	48 (26%)
Scalp tenderness	42 (23%)
Claudication of tongue	2 (1%)
Fever ≥ 38 °C	35 (19%)
Weight loss > 2 kg within 4 weeks	50 (27%)
Night sweat	33 (18%)
Polymyalgia rheumatica	90 (48%)
Biopsy of the temporal artery performed/positive*	135 (73%)/73 (54%)
MR angiography of aorta performed/positive*	170 (91%)/123 (72%)
MR angiography of extracranial arties performed/positive*	132 (71%)/63 (48%)
PET imaging performed/positive*	20 (11%)/12 (60%)
Duplex ultrasound of extracranial arteries performed/positive*	43 (23%)/18 (42%)

*% positive refers to number performed

The median age at diagnosis was 71 years. 124 (67%) patients suffered from cranial symptoms, 90 (48%) from polymyalgic symptoms. In 135 patients, temporal artery biopsies were performed, which revealed histological features of GCA in 73 (54%) patients. In 123 (72%) out of 170 performed thoracic or thoracic-abdominal MRA an aortitis was found [15].

### Vision loss

A total of 21 patients (11%) had suffered from vision loss due to GCA already prior to baseline. Unilateral vision loss had occurred in 16 patients, whereas bilateral vision loss had occurred in 5 patients prior to baseline. At baseline, best corrected visual acuity (BCVA, decimal) in the eye with acute vision loss was ≥ 0.5 in 11 eyes (42%), < 0.5 and ≥ 0.3 in 1 eye (4%), < 0.3 and ≥ 0.1 in 3 eyes (12%) and < 0.05 in 9 eyes (35%). The visual acuity at baseline of two patients was not exactly determined.

In two patients vision loss occurred while under TCZ medication: The 69-years old male patient developed AION of the left eye (BCVA of 0.01) 2 weeks after first cranial symptoms occurred and 2 days after an amaurosis fugax. The immediate treatment consisted of pulses of 1 g methyl-prednisolone over 3 days, and, in addition, one TCZ infusion (8 mg/kg body weight iv). As the AION did not improve, he received three more pulses of 500 mg methyl-prednisolone and thereafter oral prednisolone (75 mg PDN = 1 mg/kg body weight). Two weeks later, while still on 75 mg PDN daily, he lost vision of the right eye too (BCVA of 0.003).

The second patient, a woman of 78 years of age, participated in the GUSTO study (GCA treatment with Ultra-Short glucocorticoids and TOcilizumab; NCT03745586). She received three pulses of 500 mg methyl-prednisolone followed by TCZ monotherapy. Fifteen days after GC-pulse therapy she suffered from an acute vision loss (BCVA of 0), which did not improve despite immediate treatment with three additional pulses of 1 g methyl-prednisolone followed by prednisone at a dose of 1 mg/kg bodyweight.

A temporal biopsy was performed in 18 out of the 21 patients with vision loss at baseline but was negative in 6 patients (33.3%). Positive histology was more frequent in patients with vision loss compared to those without. The patients with vision loss had lower CRP levels at baseline (*p*-value of adjusted OR 0.040), were older (*p*-value 0.021), and had more often cranial symptoms (*p* value < 0.001) and jaw claudication (*p* value 0.031) and less often fever (*p* value 0.015). There was a negative association of vision loss with LVV of the aorta on MRA (*p* value 0.028) (see Tables 2 and 3).

**Table 2:** Baseline table of vision loss

	All	Normal vision at baseline	Vision loss before baseline	*p* value
	*n* (%) or median (IQ range)	*n* (%) or median (IQ range)	*n* (%) or median (IQ range)	
Total N	N = 186	*N* = 165	*N* = 21	
Female	116 (62%)	101 (61%)	15 (71%)	0.475
Age at diagnosis	71.0 (63.0; 77.0)	70.0 (63.0; 76.0)	74.0 (69.5; 82.0)	0.032
Weight [kg]	70.0 (59.0; 83.4)	71.9 (59.1; 83.3)	61.6 (54.1; 85.5)	0.178
BMI [kg/m^2^]	24.9 (22.0; 28.4)	25.2 (22.0; 28.4)	23.7 (21.3; 27.7)	0.237
First CRP (mg/L)	50.0 (20.0; 99.0)	54.5 (21.0; 101.3)	20.0 (3.5; 47.5)	0.002
First ESR (mm/h)	70.0 (40.0; 86.3)	70.0 (40.0; 87.5)	50.0 (34.0; 78.0)	0.197
ACR Criteria 1990	109 (59%)	94 (57%)	15 (71%)	0.245
Cranial symptoms (incl. Visual sympt.)	124 (67%)	103 (62%)	21 (100%)	< 0.001
Visual symptoms	70 (38%)	49 (30%)	21 (100%)	< 0.001
Permanent vision loss	21 (11%)	0 (0%)	21 (100%)	< 0.001
Headache	93 (50%)	83 (50%)	10 (48%)	1.000
Jaw claudication	48 (26%)	38 (23%)	10 (48%)	0.031
Scalp tenderness	42 (23%)	36 (22%)	6 (29%)	0.579
Claudication of tongue	2 (1%)	1 (1%)	1 (5%)	0.214
Fever ≥ 38 °C	35 (19%)	35 (21%)	0 (0%)	0.015
Weight loss > 2 kg within 4 weeks	50 (27%)	42 (25%)	8 (38%)	0.295
Night sweat	33 (18%)	29 (18%)	4 (19%)	1.000
Polymyalgia rheumatica	90 (48%)	84 (51%)	6 (29%)	0.062

**Table 3:** Adjusted OR for all the other variables

Permanent vision loss at baseline		Crude OR (95%-CI)	p-value	Adjusted OR (95%-CI)	p-value
	Age at diagnosis	1.07 (1.01 to 1.14)	0.018	1.07 (1.01 to 1.14)	0.021
	CRP (mg/L)	0.98 (0.97 to 1.00)	0.015	0.99 (0.97 to 1.00)	0.040
	Jaw claudication	3.04 (1.20 to 7.70)	0.019	2.34 (0.85 to 6.43)	0.099
	Abnormal MRA aorta	0.27 (0.10 to 0.69)	0.006	0.32 (0.12 to 0.89)	0.028

Median follow-up time of visual acuity was 17.5 (IQR 5.75–30) months. Visual acuity in the affected eyes remained stable in 15 eyes, decreased by ≥ 2 lines in 4 eyes, and increased by ≥ 2 lines in 8 eyes. One patient was lost to follow-up.

### Relapses of GCA

We identified 67/186 (36%) patients who relapsed for the first time. 20/67 (30%) patients with a first relapse had at least a 2nd relapse. The data suggests that relapses occur at a comparable rate in patients who already had a relapse and in patients who did not yet have a relapse (OR = 0.76, 95% CI 0.39;1.43; *p* value for a Fisher’s exact test (1-sided): 0.224).

25/186 (13.4%) patients with a first relapse relapsed before treatment with TCZ, 18/186 (9.7%) during treatment. Sixty-seven patients stopped TCZ during follow-up and 24/67 (35.8%) had a relapse after discontinuation of TCZ (Table 4). The relapses before start of TCZ occurred either under GC monotherapy or in combination with other conventional or biological disease-modifying anti-rheumatic drugs (DMARDs). Signs and symptoms between relapsing and non-relapsing patients did not differ significantly (Table 5).

**Table 4:** Relapses

Tocilizumab	1st relapse	
	*N* (%)	joint *p* value*
		< 0.001
**During treatment**	18/186 (9.7%)	
**Before treatment**	25/186 (13.4%)	
**After treatment**	24/67 (35.8%)	

*p value from a Pearson Chi2 test

**Table 5:** Patient characteristics by relapse

	All	No relapse	Relapse	*p* value
	*n* (%) or median (IQ range)	*n* (%) or median (IQ range)	*n* (%) or median (IQ range)	
Total *N*	N = 186	*N* = 119	*N* = 67	
Female	116 (62%)	77 (65%)	39 (58%)	0.432
Age at diagnosis	71.0 (63.0; 77.0)	71.0 (66.0; 77.0)	69.0 (62.0; 76.0)	0.174
Cranial symptoms (incl. visual imp.)	124 (67%)	81 (68%)	43 (64%)	0.629
Visual symptoms	70 (38%)	49 (41%)	21 (31%)	0.209
Vision loss	21 (11%)	15 (13%)	6 (9%)	0.630
Headache	93 (50%)	58 (49%)	35 (52%)	0.760
Jaw claudication	48 (26%)	33 (28%)	15 (22%)	0.487
Scalp tenderness	42 (23%)	23 (19%)	19 (28%)	0.201
Claudication of tongue	2 (1%)	2 (2%)	0 (0%)	0.537
Fever ≥ 38 °C	35 (19%)	20 (17%)	15 (22%)	0.435
Weight loss > 2 kg within 4 weeks	50 (27%)	29 (24%)	21 (31%)	0.389
Night sweat	33 (18%)	22 (18%)	11 (16%)	0.843
Polymyalgia rheumatica	90 (48%)	58 (49%)	32 (48%)	0.878

## Discussion

Preventing vision loss remains one of the crucial aims in GCA treatment. As vision loss is irreversible in the vast majority of patients, long-term glucocorticoid medication is still used [16]. The two RCTs investigating TCZ treatment in GCA reported only one patient with vision loss [12, 13]. In the GIACTA trial, AION occurred in the lower dose treatment arm at week 24, i.e., under TCZ s.c. bi-weekly, while the patient was on concomitant prednisone at a dose of 2 mg/day. However, the RCTs were not powered to analyze the effect of TCZ on vision loss. Furthermore, the recently established national and international patient registries cannot yet answer the question either. As we started to treat GCA with TCZ around 10 years ago, we now have the opportunity to analyze the clinical course of a large cohort of GCA patients under therapy with TCZ.

The characteristics of the patients with vision loss in our cohort correspond to the data of other studies, i.e., the patients were older, the rate of positive histology of the temporal artery was higher and the CRP levels as well as the likelihood of aortitis were lower than in patients without vision loss [6, 9, 17, 18]. Most patients were on GC treatment at initiation of TCZ therapy. The duration of GC co-medication corresponds to the periods in the cited RCTs.

A total of 22 out of 186 patients (12%) suffered from vision loss in our cohort, which is in line with the reported rates in previous publications ranging from about 2–19% [6, 10]. In two cases only, vision loss occurred while patients were treated with TCZ. These two cases merit a more detailed analysis: One occurred in the context of a current clinical study (GUSTO study; NCT03745586). AION developed 15 days after GC pulse therapy, while the patient was treated with TCZ in monotherapy. It remained non-responsive to an additional methylprednisolone pulse therapy. Notably, this patient suffered from advanced arteriosclerosis as detectable in MRA, coronary heart disease and arterial hypertension. The second patient had suffered from AION in one eye and experienced vision loss in the second, despite immediate methyl-prednisolone treatment as standard of care. In this case, a single infusion of TCZ was administered in the hope of having an additional effect. Thus, both cases presented with severe ischemic and treatment-resistant symptoms. It appears likely that structural changes were too advanced to respond to short-term, intense immunosuppression. The fact that vision loss occurred early in the disease course supports this interpretation. Furthermore, in the first patient, treatment was successfully switched back to TCZ monotherapy, after stable remission under GC therapy was achieved, thus arguing against a non-response to TCZ. Advanced structural changes of extracranial arteries are well known from MRA and from arterial biopsies. If MRA of extracranial arteries are used for diagnostic purposes, a pitfall in interpretation is the loss of the “dark blood” sign. In case of intensely inflamed arterial walls, the lumen may be obliterated, the “dark blood” signal is lost and the vessel is misdiagnosed as a vein [19]. In histology of temporal artery specimens, a fibrosis of the arterial wall together with a thickening of the intima and an obliteration of the lumen is a well-known finding. Taken together, it appears likely that a critical narrowing of arterial blood vessels due to advanced structural changes predispose to AION.

Regarding GC, most studies showed a higher percentage of vision loss during treatment. One recent case-control study with 104 GCA-patients showed new ischemic events (AION) in 4% after initiation of treatment with GC [20]. An abstract of the ACR 2019 presented a cohort of 11,820 veterans in the USA with ophthalmologic complications of 6.2% within 1 year after diagnosis despite prednisone exposure [21]. The lowest rate of vision loss under treatment with corticosteroids was reported in a cohort of 136 biopsy-proven GCA with one vision loss (0.7%) 14 months after start of treatment at a dose of 12.5 mg/d prednisolone [10]. One retrospective study found a percentage of 10% of patients with recurrent AION in the same eye during treatment with GC (3–60 mg/d at 3–36 months of treatment) [22]. In summary, the percentage of vision loss reported by our data is below or equal to the data of ophthalmological studies with high-dose GC treatment [6, 10]. As vision loss occurs at a comparable rate and at a comparable time point of disease, the same cause of AION is likely responsible in GC and TCZ treatment.

Regarding evolution of vision loss, 15/28 eyes (54%) showed a stabilization of visual acuity while 8/28 eyes (29%) showed an improvement while treated with TCZ and GC. As the analysis of data was retrospective, these findings do not represent the whole cohort. Nevertheless, they argue for a stabilization of visual impairment during therapy, which is similar to an earlier study with GC treatment only. This prospective study of 34 biopsy-proven GCA with vision loss and treatment with GC showed a deterioration of visual acuity by 2 or more lines in 27% of the patients despite GC pulse treatment 1 g iv for 3 days, followed by 60–80 mg/d and tapering of GC [7]. Another study showed an improvement in visual acuity in 5 of 39 eyes (13%) with vision loss from biopsy-proven GCA after administration of 3 iv GC-pules followed by 1 mg/kg bodyweight prednisone [23].

The relapse rate of GCA during TCZ treatment was lower in this cohort as compared to the follow-up data of the first RCT [24]. This is explained by the fact that the follow-up study reported about patients after termination of immuno-suppressive treatment. It corroborates the remission-maintaining effect of TCZ in GCA. We did not find any variables at baseline predicting relapse during therapy, whereas the follow-up data of the RCT identified younger age and more intense mural enhancement in MRA as risk factors for relapse.

Weaknesses of this study are the retrospective nature, the monocentric approach and the lack of a stringent protocol regarding GC-reduction. Furthermore, data regarding infection rate before and during or after TCZ treatment was not collected. Strengths are the sample size, the long-term data, and the meticulous data analysis of visual loss by an expert ophthalmologist.

## Conclusion

This is the first study focusing on the occurrence of vision loss in patients with GCA receiving TCZ treatment according to the protocol of the first two RCTs. Only 1 % of patients lost vision under TCZ treatment, a figure comparable to historic rates of 0.7–10% for standard GC therapy. Collectively, the data supports a central role of IL-6 and underlines the therapeutic benefit of TCZ in cranial GCA.

## Data Availability

The datasets used and/or analyzed during the current study are available from the corresponding author on reasonable request.

## Abbreviations

ACR: American College of Rheumatology
AION: Anterior ischemic optic neuropathy
BCVA: Best corrected visual acuity
CRAO: Central retinal artery occlusion
CRP: C-reactive protein
CTU: Clinical Trial Unit
DMARD: Disease-modifying antirheumatic drug
EULAR: EUropean League Against Rheumatism
ESR: Erythrocyte sedimentation rate
GC: Glucocorticoids
GCA: Giant cell arteritis
IQR: Interquartile range
LVV: Large vessel vasculitis
MRA: Magnetic resonance angiography
PDN: Prednisone
TCZ: Tocilizumab
